# Ajudecumin A from *Ajuga ovalifolia* var. *calantha* exhibits anti-inflammatory activity in lipopolysaccharide-activated RAW264.7 murine macrophages and animal models of acute inflammation

**DOI:** 10.1080/13880209.2018.1543331

**Published:** 2018-12-13

**Authors:** Hai Zhang, Qing-Cuo Ren, Yan Ren, Lin Zhao, Fan Yang, Yi Zhang, Wen-Ji Zhao, Yu-Zhu Tan, Xiao-Fei Shen

**Affiliations:** a State Key Laboratory Breeding Base of Systematic Research Development and Utilization of Chinese Medicine Resources, Sichuan Province and Ministry of Science and Technology, College of Pharmacy and College of Ethnic Medicine, Chengdu University of Traditional Chinese Medicine, Chengdu, China;; b Department of Traditional Chinese Medicine, College of Pharmacy, Southwest Minzu University, Chengdu, China;; c Key Laboratory of Birth Defects and Related Diseases of Women and Children (Ministry of Education), West China Second University Hospital Sichuan University, Chengdu, China;; d Sichuan Academy of Grassland Sciences, Chengdu, China

**Keywords:** Traditional Chinese medicine, natural products, NF-κB pathway, ERK/p38 MAPK signalling

## Abstract

**Context:** *Ajuga ovalifolia* Bur. et Franch. var. *calantha* (Diels) C. Y. Wu et C. Chen (Labiatae), a traditional Chinese medicine, has been used to treat several inflammatory diseases.

**Objective:** To assess the anti-inflammatory activity of ajudecumin A isolated from *Ajuga ovalifolia* var*. calantha*, and its possible mechanisms.

**Materials and methods:** Lipopolysaccharide (LPS, 0.5 μg/mL)-stimulated RAW264.7 macrophages were used to assess the anti-inflammatory activity of ajudecumin A (1–40 μM) *in vitro*. Nitric oxide levels were evaluated by Griess reagent. The mRNA levels of iNOS, COX-2, TNF-α, IL-1β and IL-6 were determined using qRT-PCR. Phosphorylation of ERK, JNK, p38 MAPK and IκBα were detected by western Blot. To further assess the anti-inflammatory of ajudecumin A *in vivo*, mice were oral treated with ajudecumin A (10 mg/kg) or dexamethasone (0.25 mg/kg, positive control) for 5 days before administration of carrageenan or xylene. Paw and ear edema were then measured, respectively.

**Results:** Ajudecumin A (10–40 μM) decreased LPS-induced nitric oxide production with an IC_50_ value of 16.19 μM. Ajudecumin A (20 and 40 μM) also attenuated cell spreading and formation of pseudopodia-like structures, and decreased the mRNA levels of iNOS (55.23–67.04%, *p* < 0.001), COX-2 (57.58–70.25%, *p* < 0.001), TNF-α (53.75–58.94%, *p* < 0.01–0.001), IL-1β (79.41–87.85%, *p* < 0.001) and IL-6 (54.26–80.52%, *p* < 0.01–0.001) in LPS-activated RAW264.7 cells. Furthermore, ajudecumin A suppressed LPS-induced phosphorylation of ERK, p38 MAPK, and IκBα, as well as IκBα degradation (*p* < 0.05–0.001). Finally, ajudecumin A (10 mg/kg) attenuated carrageenan- and xylene-induced inflammation in mice by about 28 and 24%, respectively.

**Discussion and conclusions:** Ajudecumin A exhibited a potent anti-inflammatory activity *in vitro* and *in vivo* through inhibition on NF-κB and ERK/p38 MAPK pathways, suggesting that ajudecumin A may be potentially developed as a lead compound in anti-inflammatory drug discovery.

## Introduction

Inflammation, the most primitive protective response to a variety of stimuli, is induced and regulated by a series of immune cells (Tracey 2002). Macrophages, a highly plastic group of innate immune cells, play pivotal roles in immune responses and inflammation by producing many kinds of pro-inflammatory cytokines, inducible synthase and inflammatory mediators, including interleukin-1β (IL-1β), IL-6, tumour necrosis factor-α (TNF-α), inducible nitric oxide synthase (iNOS), cyclooxygenase-2 (COX-2) and nitric oxide (NO), etc. (Wynn et al. 2013). Furthermore, several signalling cascades such as nuclear factor κB (NF-κB) signalling pathway, and mitogen-activated protein kinases (MAPKs) signalling pathway, are activated and involved in macrophages-mediated inflammation (Huang et al. 2010; Hoesel and Schmid 2013). However, excessive activation of aforementioned pro-inflammatory signalling and overproduction of these pro-inflammatory factors in macrophages is responsible for many inflammatory diseases, such as rheumatoid arthritis, cancer, atherosclerosis, diabetes and Alzheimer disease (McNelis and Olefsky 2014; Heppner et al. 2015). Therefore, regulating the crucial proteins in these inflammatory signalling pathways or inhibiting the production of pro-inflammatory factors may serve to prevent or suppress a variety of inflammatory diseases (Pandurangan et al. 2016; Alvarez-Suarez et al. 2017; Xu et al. 2017).

**Figure 1.**
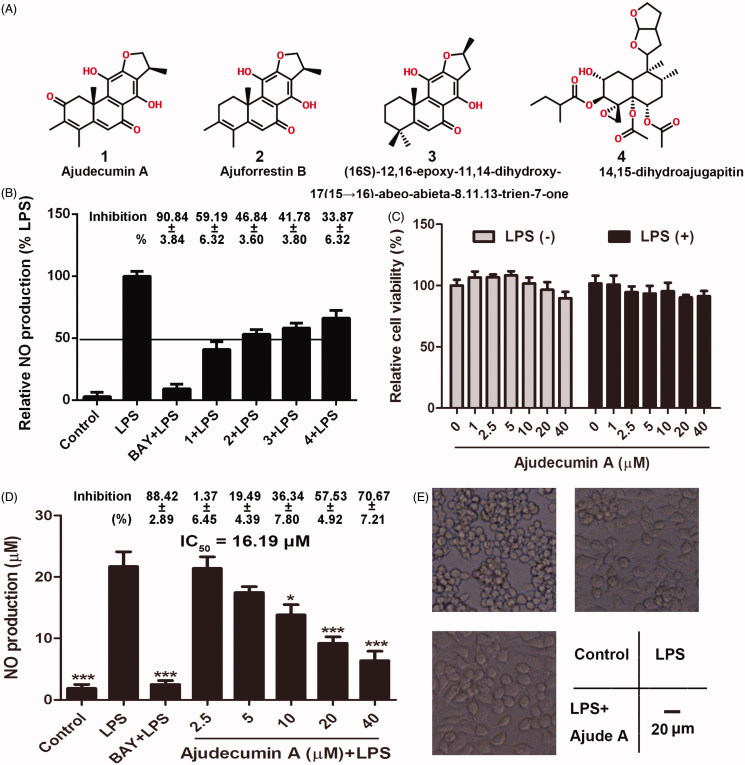
Ajudecumin A inhibited NO production and morphological changes in LPS-activated RAW264.7 macrophages. (A) Chemical structure of Ajudecumin A (**1**), Ajuforrestin B (**2**), 14,15-dihydroajugapitin (**3**), and (16*S*)-12,16-epoxy-11,14- dihydroxy-17(1 5 →16)-abeo-abieta-8,11,13-trien-7-one (**4**) from *Ajuga ovalifolia* var. *calantha*. (B and D) RAW264.7 cells were pre-treated with the tested compounds at the indicated concentrations and BAY 11-7082 (5 μM) for 2 h, and then incubated with LPS for 24 h. Levels of NO in the culture medium were assessed using Griess reagent. (C) Cells were treated with the tested compounds at the indicated concentrations for 24 h in the presence of LPS (0.5 μg/ml) or not. Cell viability was measured by CCK8 assay and expressed relative to the vehicle control. All data are represented as mean ± SD, *n* = 6. *p < 0.05, **p < 0.01, ***p < 0.001 vs. LPS alone. The inhibition rates (%, compared with the LPS alone) are also shown. (E) After treatment of Ajudecumin A and LPS, the morphology of RAW264.7 cells was observed under light microscope, bar =20 μm.

The genus *Ajuga* (Labiatae) is distributed over the Eurasian continent. Several species of this genus have been reported to be rich sources of bioactive metabolites, including diterpenes, steroids and iridoids, which exhibit insect antifeedant, antimicrobial, cytotoxic and vasoconstrictor activities (Israili and Lyoussi 2009; Qing et al. 2017). The whole plant of *Ajuga ovalifolia* Bur. et Franch. var. *calantha* (Diels) C. Y. Wu et C. Chen (Labiatae) is used in folk medicine in China for the treatment of inflammation (Guo et al. 2011a, 2011b, 2012). Phytochemical studies showed that diterpenes are the main bioactive constituents in *Ajuga*. Recently, some new clerodane diterpenoids isolated form *Ajuga* have been regarded as neuroprotective agents against 1-methyl-4-phenyl-1,2,3,6-tetrahydropyridine (MPP+)-induced SH-SY5Y neuronal cell death and lipopolysaccharide (LPS)-induced inflammation in microglial BV-2 cells (Guo et al. 2011a, 2011b, 2012). However, pharmacological and mechanism studies on *Ajuga* and its bioactive components are limited. Previously, we isolated four diterpenes, including ajudecumin A (**1**), ajuforrestin B (**2**), (16*S*)-12,16-epoxy-11,14-dihydroxy-17(1 5 →16)-abeo-abieta-8,11, 13-trien-7-one (**3**) and 14,15-dihydroajugapitin (**4**) (Figure 1(A)) from *Ajuga ovalifolia* var. *calantha* (Chen et al. 2017b). Among these compounds, ajudecumin A exhibited moderate inhibitory activity on the proliferation of human breast cancer MCF-7 cells (Wang et al. 2012); 14,15-dihydroajugapitin showed an antibacterial activity against *Escherichia coli* (Ganaie et al. 2017). Diterpenes are known for their biological and pharmacological characteristics, such as antibacterial, anticancer and anti-inflammatory activities (Tran et al. 2017). In the present study, we further evaluated the anti-inflammatory activity and underlying mechanism of these four diterpenes in LPS-activated murine RAW264.7 macrophage cells, as well as carrageenan- and xylene- induced acute inflammation models.

## Materials and methods

### Reagents

The four diterpenes, including ajudecumin A (**1**), Ajuforrestin B (**2**), (16*S*)-12,16-epoxy-11,14-dihydroxy-17(1 5 →16)-abeo-abieta-8,11,13-trien-7-one (**3**), and 14,15-dihydroajugapitin (**4**) were isolated from *Ajuga ovalifolia var. calantha* and identified by NMR (Chen et al. 2017b), (Supplementary Figures S1 and S2). LPS from *Escherichia coli* 055:B5 and carrageenan were provided by Sigma (Shanghai, China). BAY 11-7082, CCK-8 agent, Griess agent was obtained from Beyotime (Haimen, China). Antibody against iNOS is the products of Cell Signalling Technology (Danvers, USA). Phospho- and total-ERK, phosphor- and total-p38 MAPK, phosphor- and total-JNK, phosphor- and total-IκBα antibodies were purchased from Signalway Antibody (Baltimore, USA). COX-2 and actin antibodies as well as HRP-conjugated secondary antibody were from Proteintech (Wuhan, China).

### Cell culture

Murine macrophage RAW264.7 cells were provided by the Cell Bank of the Chinese Academy of Sciences (Shanghai, China). RAW264.7 cells were cultured in Dulbecco’s Modified Eagle Medium (DMEM) supplemented with 10% foetal bovine serum (FBS) and 1% penicillin/streptomycin (Hyclone, Beijing, China) in a humidified incubator with a 5% CO_2_ atmosphere at 37 °C.

### Animal

Male Kunming (KM) mice (about 6 weeks, and 22 g) were purchased from Chengdu Dashuo Biological Company (Chengdu, China). Animals were kept in plastic cages at 25 ± 1 °C with free access to pellet food and water and on a 12 h light/dark cycle. Animal welfare and experimental procedures were strictly adhered to, in accordance with the Guide for the Care and Use of Laboratory Animals published by the US National Institutes of Health (NIH Publication No. 85-23, revised 1996). The experimental scheme of animal study was approved by the ethics committee of Chengdu University of Traditional Chinese Medicine (No. 2018-05).

### Cell viability assay

Cell viability was assessed by CCK8 assay. In brief, RAW264.7 cells were seeded into 96-well plates at a density of 2.5 × 10^4^ cells/well, and incubated at 37 °C overnight. Cells were treated with different concentrations of ajudecumin A (1, 2.5, 5, 10, 20 and 40 μM) for 24 h in presence of LPS (0.5 μg/mL). Next, cells were incubated with 10 μL of CCK-8 for 2 h at 37 °C. Subsequently, absorbance at 562 nm was read using a scanning microtiter apparatus (Thermo Fisher Scientific, Waltham, USA). Relative cell viability was defined as the ratio of the absorbance in test wells compared to control wells.

### Determination of nitric oxide (NO)

Briefly, RAW264.7 cells were seeded into a 24-well plate at a density of 2.5 × 10^5^ cells/well, incubated overnight, and pre-treated with the four compounds (20 μM) mentioned above or different concentrations of ajudecumin A (2.5, 5, 10, 20 and 40 μM) for 2 h, followed by stimulation with LPS (0.5 μg/mL) for an additional 24 h. Levels of NO in cell culture medium were evaluated by Griess reaction.

### Quantitative real-time polymerase chain reaction (qRT-PCR)

RAW264.7 cells (2 × 10^6^ cells/well) were plated in 6-well plate, incubated overnight, and treated with different concentrations of ajudecumin A and BAY 11-7082 for 2 h, followed by treatment with LPS for an additional 24 h. Total RNAs were extracted using a UNlQ-10 Column total RNA Purification Kit (Sangon Biotech, Shanghai, China), and then were reverse-transcribed to cDNAs by using All-in-One cDNA Synthesis SuperMix Kit (Bimake, Shanghai, China) according to the manufacturer’s protocol. qRT-PCR was performed on a CFX96 Real-Time PCR System (Bio-Rad, Hercules, CA, USA) with SYBR Green (Bimake, Shanghai, China). Relative expression levels of the target genes were calculated based on 2^−ΔΔCt^ according to the manufacturer’s specifications by using the GAPDH gene as a reference gene. The primers were used as follows (Zhao et al. 2018). TNF-α forward primer: 5′-CAC CAC GCT CTT CTG TCT-3′, TNF-α reverse primer: 5′-GGC TAC AGG CTT GTC ACT C-3′; IL-1β forward primer: 5′-CAA CCA ACA AGT GAT ATT CTC CAT G-3′, IL-1β reverse primer: 5′-GAT CCA CAC TCT CCA GCT GCA-3′; IL-6 forward primer: 5′-TAG TCC TTC CTA CCC CAA TTT CC-3′, IL-6 reverse primer: 5′-TTG GTC CTT AGC CAC TCC TTC-3′; iNOS forward primer: 5′-CCT GTG AGA CCT TTG ATG-3′, iNOS reverse primer: 5′-CCT ATA TTG CTG TGG CTC-3′; COX2 forward primer: 5′-CAA CAC CTG AGC GGT TAC-3′, COX2 reverse primer: 5′-GTT CCA GGA GGA TGG AGT-3′; GAPDH forward primer: 5′-TGC ACC ACC AAC TGC TTA GC-3′, GAPDH reverse primer: 5′-GGC ATG GAC TGT GGT CAT GAG-3′.

### Western blot assay

After treatment, RAW264.7 cells were lysed with RIPA buffer in the presence of proteases and phosphatases inhibitors (Beyotime, Haimen, China). The cell lysates were subjected to SDS-PAGE. Then, the proteins were blotted onto the PVDF membrane (Millipore, Bedford, USA). The membrane was blocked with 5% BSA in TBST buffer, and incubated with specific primary antibodies overnight at 4 °C. After washing, the membrane was incubated with HRP-conjugated secondary antibody for 1 h at room temperature, signals were then detected by chemiluminescence (Beyotime, Haimen, China), and quantified by using QuantityOne.

### Anti-inflammatory evaluation in mice

The carrageenan-induced paw edema and xylene-induced ear edema were used to evaluate the anti-inflammatory activity of ajudecumin A *in vivo* (Shen et al. 2017; Zhao et al. 2018). Briefly, KM mice were pre-treated with ajudecumin A (10 mg/kg), dexamethasone (0.25 mg/kg, positive control) and vehicle for 5 days, respectively. After 1 h of the last administration, mice were intradermally injected with 1% w/v carrageenan into the right hind paw. Paw volume was measured by a plethysmometer at 0 and 4 h after carrageenan injection. For xylene-induced ear edema, mice were pre-treated with dexamethasone and ajudecumin A for 5 days. One hour after the last administration, the surface of the right ear was smeared with 20 μL of xylene to induce ear edema. The left ear was considered as control. One hour later, the mice were sacrificed by cervical dislocation; circular sections of the right and left ears were then taken with a cork borer (diameter of 7 mm) and weighed. Finally, the inflamed paws and ears were removed, and fixed with 4% paraformaldehyde. Tissues were then dehydrated, processed, embedded in paraffin, sectioned and stained with haematoxylin and eosin (HE). The sections were examined under a light microscope and photographs were taken.

## Results and discussion

### Ajudecumin a inhibited the production of NO and morphological changes in LPS-activated RAW264.7 macrophages

Diterpenes are known for their biological properties, such as anti-inflammatory activity (Tran et al. 2017). We thus assessed the anti-inflammatory activities of four diterpenes from *Ajuga ovalifolia* var. *calantha*. NO, a well-known biomarker of inflammatory response induced by numerous inflammatory stimulation, contributes to tissue damage in various inflammatory and autoimmune diseases at high concentrations (Bogdan, 2001). Thus, NO is a valuable indicator in the screening of anti-inflammatory agents (Alvarez-Suarez et al. 2017; Xu et al. 2017). In this study, LPS, a component of the Gram-negative bacteria cell wall, was used to induce inflammatory response in macrophages through promoting the release of various inflammatory factors, including NO (Xu et al. 2017). As expected, LPS significantly induced NO production, and pre-treatment with BAY 11-7082 (5 μM), a known IκB inhibitor, repressed the overproduction of NO. Furthermore, the four diterpenes from *Ajuga ovalifolia* var. *calantha* also could inhibit LPS-induced NO production at concentration of 20 μM. Compared with compound 2, 3 and 4, compound 1 (ajudecumin A) showed the strongest anti-inflammatory activity in LPS-activated RAW264.7 cells (Figure 1(B)). Hence, ajudecumin A was selected to further evaluate the effect and mechanism on inflammatory response *in vitro* and *in vivo*.

To exclude the possibility that any anti-inflammatory activity or related events in the following study were attributed to the cytotoxicity of ajudecumin A on RAW 264.7 macrophages, the cells were pre-treated with a series of concentrations of ajudecumin A for 2 h and then cultured with or without LPS (0.5 μg/mL) for additional 24 h. As shown in Figure 1(A), no significant cytotoxicity of ajudecumin A in RAW264.7 cells were observed at the tested concentrations up to 40 μM. Therefore, the concentrations ranging from 1 to 40 μM for ajudecumin A were used in the next assay.

Furthermore, we found that ajudecumin A obviously suppressed the NO production in a concentration-dependent manner in LPS-stimulated RAW 264.7 cells (Figure 1(B)), and the value of IC_50_ is 16.19 μM. Additionally, the unstimulated macrophages generally display spear and smooth shaped forms. Upon exposing to various inflammatory stimuli, such as LPS, the macrophages display an activated phenotype, which mainly manifested as irregular and rough form with accelerated spreading and pseudopodia-like formations (Purushotham et al. 2017). As expected, LPS caused irregular and rough form with accelerated spreading in RAW264.7 cells, which can be obviously improved by ajudecumin A treatment (Figure 1(C)). Taken together, ajudecumin A suppressed the overproduction of NO and morphological changes in LPS-activated RAW264.7 macrophages, which suggesting the anti-inflammatory activity of ajudecumin A.

### Ajudecumin a decreased the mRNA and protein levels of iNOS and COX-2 in LPS-activated RAW264.7 macrophages

**Figure 2.**
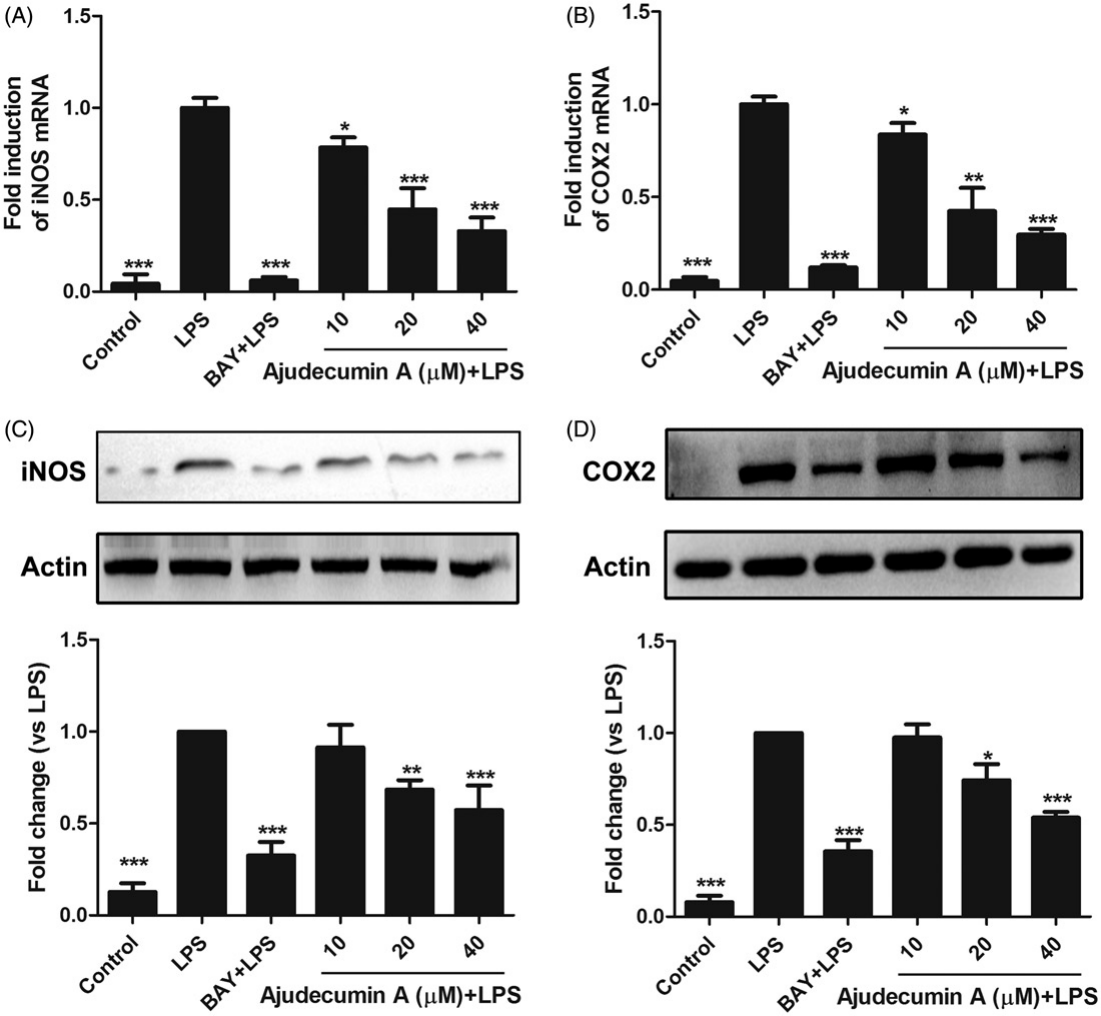
Ajudecumin A inhibited the mRNA and protein expression of iNOS and COX2 in LPS-stimulated RAW264.7 cells. Cells were pre-treated with indicated concentrations of Ajudecumin A and BAY 11-7082 (5 μM) for 2 h before the addition of LPS (0.5 μg/ml) for 24 h. The mRNA levels of iNOS (A) and COX2 (B) were measured by qRT-PCR with GAPDH used as an internal control. All data are represented as mean ± SD, *n* = 6. (C) Cell lysates were immunoblotted with antibodies against iNOS and COX2. Actin staining is shown as a loading control. Representative image of three independent experiments are shown, and the quantitative results are depicted. All data are represented as mean ± SD, *n* = 3. *p < 0.05, **p < 0.01, ***p < 0.001 vs. LPS control.

The production of NO is tightly regulated by iNOS, which is expressed predominantly in activated macrophages (Bogdan 2015). To determine whether the inhibition of NO production by ajudecumin A is attributed to its ability in inhibiting the expression of iNOS, we performed RT-qPCR and Western Blot assay to detect the mRNA and protein levels of iNOS, respectively. As a control, the IκB inhibitor BAY11-7082 remarkably decreased iNOS expression by 93.90 and 67.47% at both mRNA and protein levels (Figure 2(A,C)), consistent with the result that iNOS was tightly regulated by the NF-κB signalling. Ajudecumin A at doses of 10, 20 and 40 μM also suppressed the mRNA expression of iNOS by 21.57, 55.23 and 67.04% (*p* < 0.05–0.001), respectively, compared with that in LPS-treated RAW264.7 cells (Figure 2(A)). Consistent with its effects on iNOS transcription, iNOS protein expression was decreased by treatment with ajudecumin A (20 and 40 μM, the inhibition rate were 31.57 and 45.96%, respectively, *p* < 0.01–0.001, Figure 2(C). These results indicated that ajudecumin A inhibited NO production in LPS-activated RAW264.7 cells by suppression of iNOS expression.

COX-2 is another important inducible enzyme that amplificates inflammatory responses through catalyzing the rate-limiting step in the synthesis of prostaglandins (PGs) (Cha and DuBois 2007). Furthermore, the elevated expression and activity of COX-2 is always observed in a series of inflammatory cells, including macrophages (Dennis and Norris 2015). Therefore, COX-2 is considered as a promising target for the treatment of inflammatory diseases. We thus determined whether ajudecumin A affects COX-2 expression. As a potent inflammatory stimulator, LPS induced a remarkable elevation in mRNA and protein expression of COX-2 in RAW264.7 cells; and this abnormal elevation was attenuated by ajudecumin A treatment (40 μM, the inhibition rate on mRNA and protein levels were 70.25 and 46.07%, respectively, *p* < 0.001, Figure 2(B,D). This result indicated that ajudecumin A exerted an anti-inflammatory effect, in part, by inhibition of COX-2 expression.

### Ajudecumin a suppressed the mRNA level of pro-inflammatory cytokines in LPS-stimulated RAW264.7 cells

**Figure 3.**
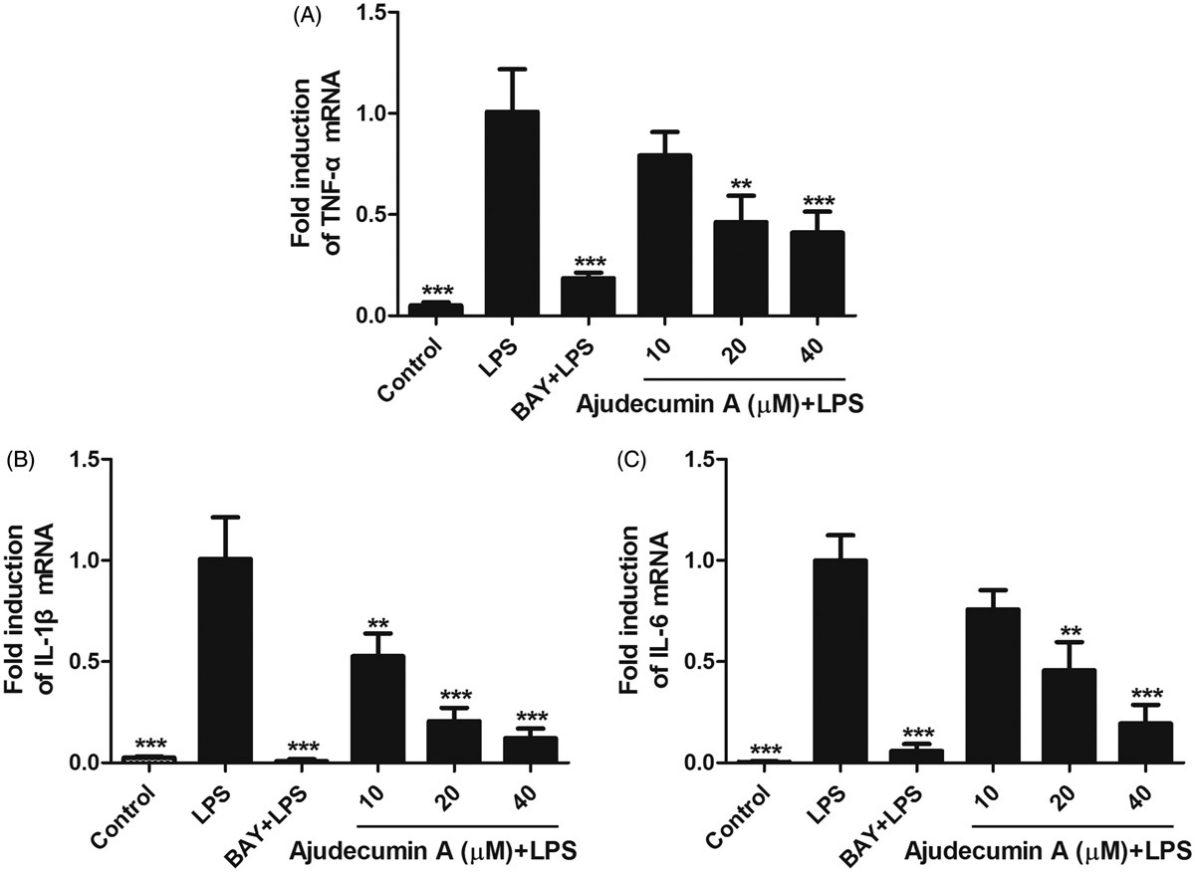
Ajudecumin A suppressed the mRNA expression of pro-inflammatory cytokines in LPS-stimulated RAW264.7 cells. Cells were pre-treated with various concentrations of Ajudecumin A and BAY 11-7082 (5 μM) for 2 h, and then cultured with LPS (0.5 μg/mL) for 24 h. The mRNA levels of TNF-α (A), IL-1 β (B), and IL-6 (C) were measured by qRT-PCR with GAPDH used as an internal control. All data are represented as mean ± SD, *n* = 6. *p < 0.05, **p < 0.01, ***p < 0.001 vs. LPS control.

In addition to the inducible enzymes and inflammatory mediators, LPS expose also results in the production of several pro-inflammatory cytokines such as TNF-α, IL-1β and IL-6 (Mosser and Edwards 2008). To further address the anti-inflammatory activity of ajudecumin A, we thus evaluated whether it can alter the mRNA expression of TNF-α, IL-1β and IL-6 in LPS-stimulated RAW264.7 cells. As shown in Figure 3(A,C), treatment with ajudecumin A (20 and 40 μM) diminished the TNF-α and IL-6 mRNA expression by 53.75 and 58.94%, 54.26 and 80.52%, respectively (*p* < 0.01–0.001). Likewise, compared to the vehicle control, treatment with 10, 20 and 40 μM of ajudecumin A also caused 47.28, 79.41 and 87.85% decrease in IL-1β gene expression in LPS-treated RAW264.7 cells, respectively (*p* < 0.01–0.001, Figure 3(B)).

Synthesis and release of pro-inflammatory cytokines in macrophages by stimuli is a crucial step in the initiation and amplification of inflammation (Mosser and Edwards 2008). Among them, TNF-α and IL-6 can bind to their receptors, thereby triggering downstream activation of inflammatory gene expression, and are responsible for a series of inflammatory disorders, including rheumatoid arthritis and inflammatory bowel disease (Hodge et al. 2005; Billiet et al. 2014). Similarly, IL-1β, an early major pro-inflammatory cytokine mediating the inflammatory response at both the local and systemic levels, is also strongly involved in some autoimmune diseases, such as rheumatoid arthritis (Palomo et al. 2015). Thus, inhibition of these pro-inflammatory cytokines is conducive to the treatment of inflammatory diseases and has become a potential target for novel anti-inflammatory drugs. The present study has demonstrated that ajudecumin A can efficiently attenuate the production of pro-inflammatory cytokines at the transcriptional level.

### Ajudecumin a suppressed IκBα phosphorylation and degradation in LPS-activated RAW264.7 macrophages

NF-κB, a pleiotropic transcription factor, plays a crucial role in inflammation triggering and amplifying through up-regulating the expression of multiple genes, such as pro-inflammatory cytokines, chemokines and inducible enzymes (Durand and Baldwin 2017). Thus, NF-κB has been considered as an attractive drug target for anti-inflammatory therapy (Killeen et al. 2014). Normally, NF-κB displays an inactive form in the cytoplasm by binding to its inhibitor protein of IκB. Upon stimulation by LPS or pro-inflammatory cytokines, the IκB protein can be rapidly phosphorylated by IκB kinase, thereby triggering a proteasome-mediated degradation, which is conducive to NF-κB activation. The activated NF-κB is then translocated into the nucleus, where it induces the expression of multiple inflammatory genes by binding to the specific sequences of DNA (Hoesel and Schmid 2013). Herein, we thus further determine whether the inhibitory action of ajudecumin A on pro-inflammatory cytokines and inducible enzymes was due to its inhibition of IκBα phosphorylation and degradation.

**Figure 4.**
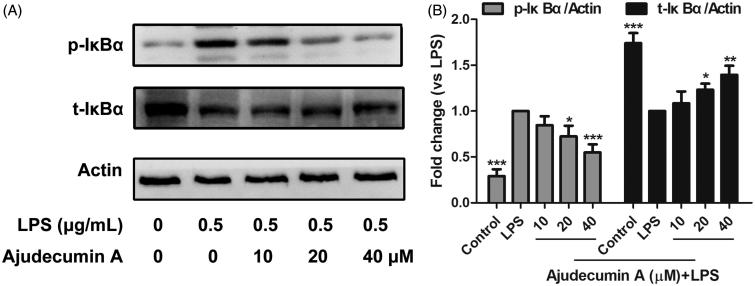
Ajudecumin A blocked the activation of NF-κB pathway in LPS-activated RAW264.7 macrophages. (A) Cells were pre-treated with Ajudecumin A (10, 20, and 40 μM) for 4 h before the addition of LPS (0.5 μg/mL) for 5 min. The IκBα phosphorylation and degradation were evaluated by western Blot. Actin is shown as a loading control. Results from representative experiments are shown, and the quantitative results are depicted (B). All data are represented as mean ± SD, *n* = 3. *p < 0.05, **p < 0.01, ***p < 0.001 vs. LPS control.

As shown in Figure 4(A,B), in untreated cells, LPS stimulation caused a significant increase of IκBα phosphorylation and reduction in IκBα protein level, which was consistent with the previous finding that LPS stimulation can trigger IκBα degradation (Akira and Takeda 2004). Treatment of ajudecumin A at doses of 20 and 40 μM decreased LPS-induced IκBα phosphorylation in RAW264.7 cells by about 27.51% and 45.07% when compared with LPS control (*p* < 0.05–0.001, Figure 4(A,B)). Similarly, 20 and 40 μM of ajudecumin A also obviously elevated IκBα protein level in LPS-stimulated RAW264.7 cells by about 23.30% and 39.57% when compared with vehicle control (*p* < 0.05–0.001, Figure 4(A,B)). These findings suggested that ajudecumin A exerted an anti-inflammatory action, in part, through suppression on the LPS-activated NF-κB signalling pathway.

### Ajudecumin A attenuated the phosphorylation of ERK and p38 MAPK in LPS-stimulated RAW264.7 cells

The MAPK superfamily proteins, including ERK, p38 MAPK and JNK, plays a key role in regulating the secretion of pro-inflammatory cytokines, mediators, and inducible enzymes in activated macrophages and other cell types (Kim and Choi 2015). Previous work reported that activated MAPKs are found in several inflammatory diseases (Thalhamer et al. 2008). These findings emphasize MAPKs as potential therapeutic targets in some inflammatory diseases. Furthermore, several studies have indicated that some natural compounds including resveratrol and curcumin exert their anti-inflammatory activity through regulating the MAPKs signalling pathway (Koeberle and Werz 2014).

**Figure 5.**
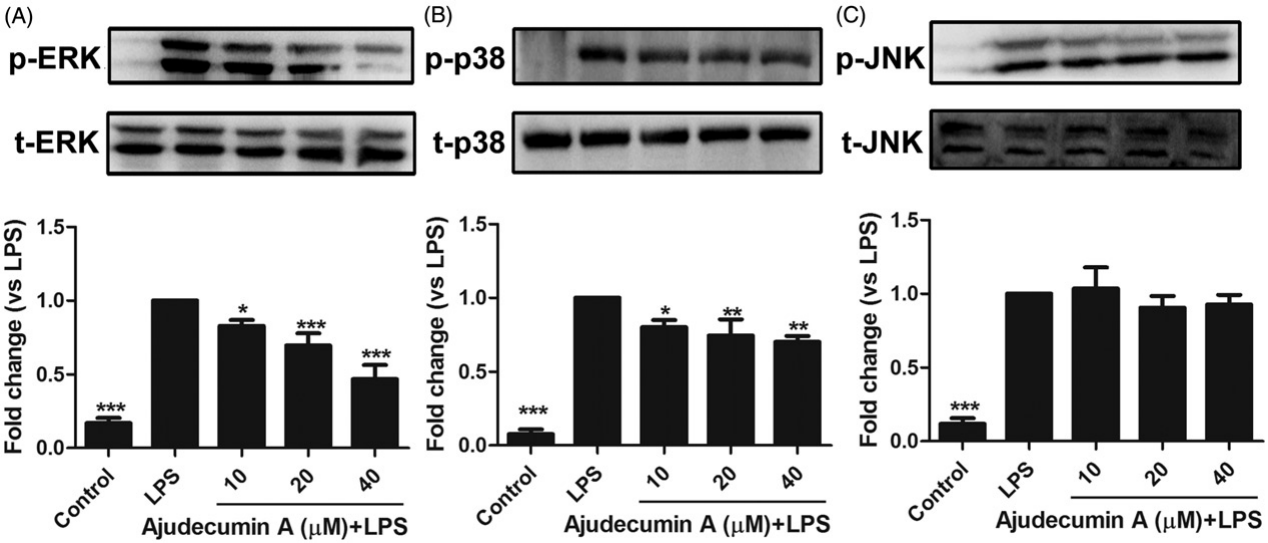
Ajudecumin A repressed the phosphorylation of ERK and p38 MAPK in LPS-stimulated RAW264.7 cells. RAW 264.7 cells were pre-incubated with indicated concentrations of Ajudecumin A for 4 h and then stimulated with 0.5 μg/mL of LPS for another 30 min. An equal amount of protein sample was used to measure the phosphorylation of ERK (A), p38 MAPK (B), and JNK (C) using specific antibodies. The level non-phosphorylated MAPKs protein was used as the internal control. Results from representative experiments are shown, and the quantitative results are depicted. All data are represented as mean ± SD, *n* = 3. *p < 0.05, **p < 0.01, ***p < 0.001 vs. LPS control.

To explore whether MAPKs signalling is involved in ajudecumin A-mediated anti-inflammatory action in LPS-activated RAW264.7 cells, we assessed the phosphorylation levels of ERK, p38 MAPK, and JNK were via Western Blot. As displayed in Figure 5(A–C), stimulation of LPS significantly enhanced the phosphorylation of ERK, p38 MAPK, and JNK in RAW264.7 cells. In contrast, LPS-induced activation of ERK was attenuated by ajudecumin A treatment in a concentration-dependent manner (10, 20 and 40 μM, inhibition rate were 17.07, 30.50 and 53.26%, respectively, *p* < 0.05–0.001, Figure 5(A). Likewise, compared with vehicle control, ajudecumin A also could inhibit the increased phosphorylation of p38 MAPK by 19.77, 25.50 and 29.08%, respectively (*p* < 0.05–0.01); but its inhibitory action was relatively moderate (Figure 5(B). Unlike ERK and p38 MAPK, LPS-induced phosphorylated JNK could not be clearly altered by ajudecumin A (*p* > 0.05). This result indicated an important repressor effect of ajudecumin A on activation of ERK and p38 MAPK in LPS-stimulated RAW264.7 cells, which may contribute to its anti-inflammatory effect. Moreover, ERK and p38 MAPK are known to regulate the NF-κB signalling pathway through activating the mitogen- and stress-activated kinase (MSK) protein (Saklatvala 2004; Vermeulen et al. 2009). Therefore, ajudecumin A may repress the LPS-induced NF-κB pathway activation through inhibition on the phosphorylation of ERK and p38 MAPK.

### Ajudecumin A alleviated carrageenan-induced paw edema and xylene-induced ear edema in mice

**Figure 6.**
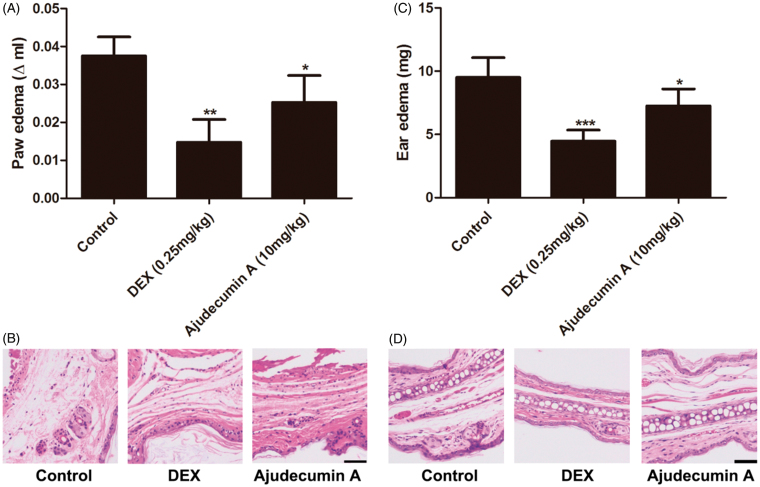
Ajudecumin A alleviated acute inflammation *in vivo*. Mice were treated with Ajudecumin A (10 mg/kg) and dexamethasone (0.25 mg/kg) by intraperitoneal injection for 5 days. Carrageenan-induced paw edema (A) and xylene-induced ear edema (C) were used to assess the anti-inflammatory effect of Ajudecumin A *in vivo*. The HE staining was used to evaluate the degree of inflammatory reaction in paw (B) and ear tissues (D). All data are represented as mean ± SD, *n* = 6. *p < 0.05, ***p < 0.001 vs. control. Bar =50 μm.

To further evaluate the anti-inflammatory activity of ajudecumin A *in vivo*, we established carrageenan-induced paw edema and xylene-induced ear edema in mice. Carrageenan is a strong pro-inflammatory agent that is used to stimulate the release of several pro-inflammatory mediators, such as prostaglandins, leukotrienes, histamine and TNF-α. Xylene-induced ear edema is mainly associated with the release of some pro-inflammatory mediators, including substance P, prostaglandins, histamine. Furthermore, the carrageenan- and xylene-induced acute inflammatory response is mainly characterized by the exudation of fluid and plasma proteins with a high degree of reproducibility (Vazquez et al. 2015). Thus, these two models are considered suitable for evaluating the effects of anti-inflammatory agents (Yoon et al. 2017; Chen et al. 2017a). As depicted in Figure 6(A), treatment with ajudecumin A at 10 mg/kg or dexamethasone at 0.25 mg/kg for 5 days significantly ameliorated carrageenan-induced acute paw edema in mice by about 28% and 63% when compared with vehicle control (*p* < 0.05–0.01), respectively. Furthermore, after 5 days treatment of ajudecumin A (10 mg/kg) and dexamethasone (0.25 mg/kg) decreased xylene-induced ear edema in mice by about 24% and 53% when compared with vehicle control (*p* < 0.05–0.001, Figure 6(C)), respectively. Meanwhile, histologic evaluation showed that the carrageenan- and xylene-induced edema, hyperaemia, and inflammatory cell infiltration could be lessened by treatment of ajudecumin A or dexamethasone (Figure 6(B,D)). The inhibitory action of ajudecumin A on carrageenan- and xylene-induced acute inflammation may be related to the inhibition of pro-inflammatory mediators.

## Conclusions

Ajudecumin A from *Ajuga ovalifolia* var. *calantha* possesses anti-inflammatory activity in LPS-activated RAW264.7 murine macrophages, and carrageenan- and xylene-induced acute inflammation in mice. All these actions may be attributed to its inhibition on NF-κB and ERK/p38 MAPK signalling. Furthermore, these results may also explain the anti-inflammatory activity of *Ajuga* reported previously, and suggesting that ajudecumin A may be an important bioactive ingredient in *Ajuga*. Finally, these findings provide additional pharmacological information and may contribute for the further study and use of ajudecumin A as a phytomedicine.

## Supplementary Material

Supplementary Figures S1 and S2.doc
