# Supplementary material for: Ajudecumin A from Ajuga ovalifolia var. calantha exhibits anti-inflammatory activity in lipopolysaccharide-activated RAW264.7 murine macrophages and animal models of acute inflammation
Source: Pharm Biol. 2018 Dec 13;56(1):649–57. doi: 10.1080/13880209.2018.1543331 (PMC7011979; doi:10.1080/13880209.2018.1543331)
Supplement: Supplementary Figures S1 and S2.doc [file IPHB_A_1543331_SM6820.doc]

**
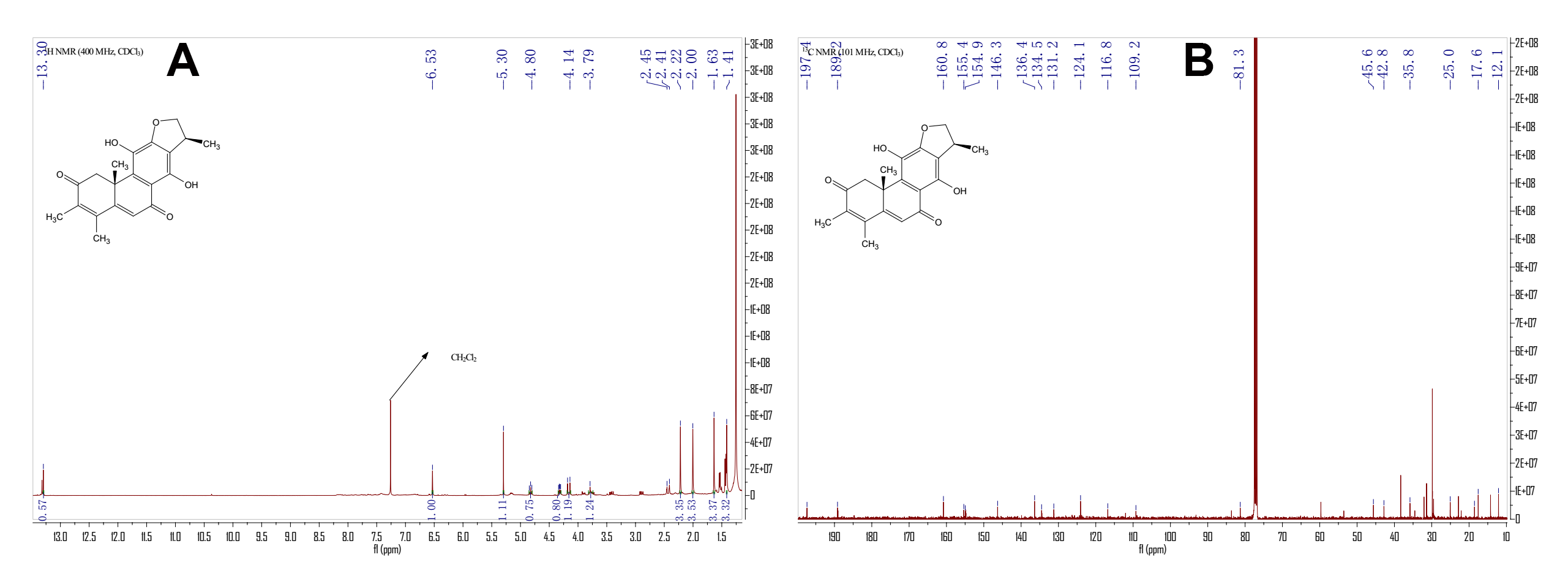
**

**Figure S1.** NMR identification of Ajudecumin A. (A) ^1^H NMR; (B) ^13^C NMR. Ajudecumin A, red powder,^1^H-NMR (400 MHz,CDCl_3_) *δ*: 13.30 (1H, brs, 14-OH), 6.53 (1H, s, H-6), 4.83 (1H, dd, *J* = 8.8, 8.8 Hz, H-16b), 4.32 (1H, dd, *J* = 8.8, 5.9 Hz, H-16a), 4.16 (1H, d, *J* = 16.7 Hz, H-1b), 3.78 (1H, m, H-15), 2.43 (1H, d, *J* = 16.7 Hz, H-1a), 2.22 (3H, s, H-19), 2.01 (3H, s, H-18), 1.64 (3H, s, H-20), 1.44 (3H, d, *J* = 6.9 Hz, H-17); ^13^C NMR (100 MHz, CDCl_3_) *δ*: 197.4 (C-2), 189.2 (C-7), 160.8 (C-5), 155.4 (C-14), 154.9 (C-12), 146.3 (C-4), 136.4 (C-3), 134.5 (C-9), 131.2 (C-11), 124.1 (C-6), 116.8 (C-13), 109.2 (C-8), 81.3 (C-16), 45.6 (C-1), 42.8 (C-10), 35.8 (C-15), 25.0 (C-20), 18.6 (C-17), 17.6 (C-19), 12.1 (C-18).


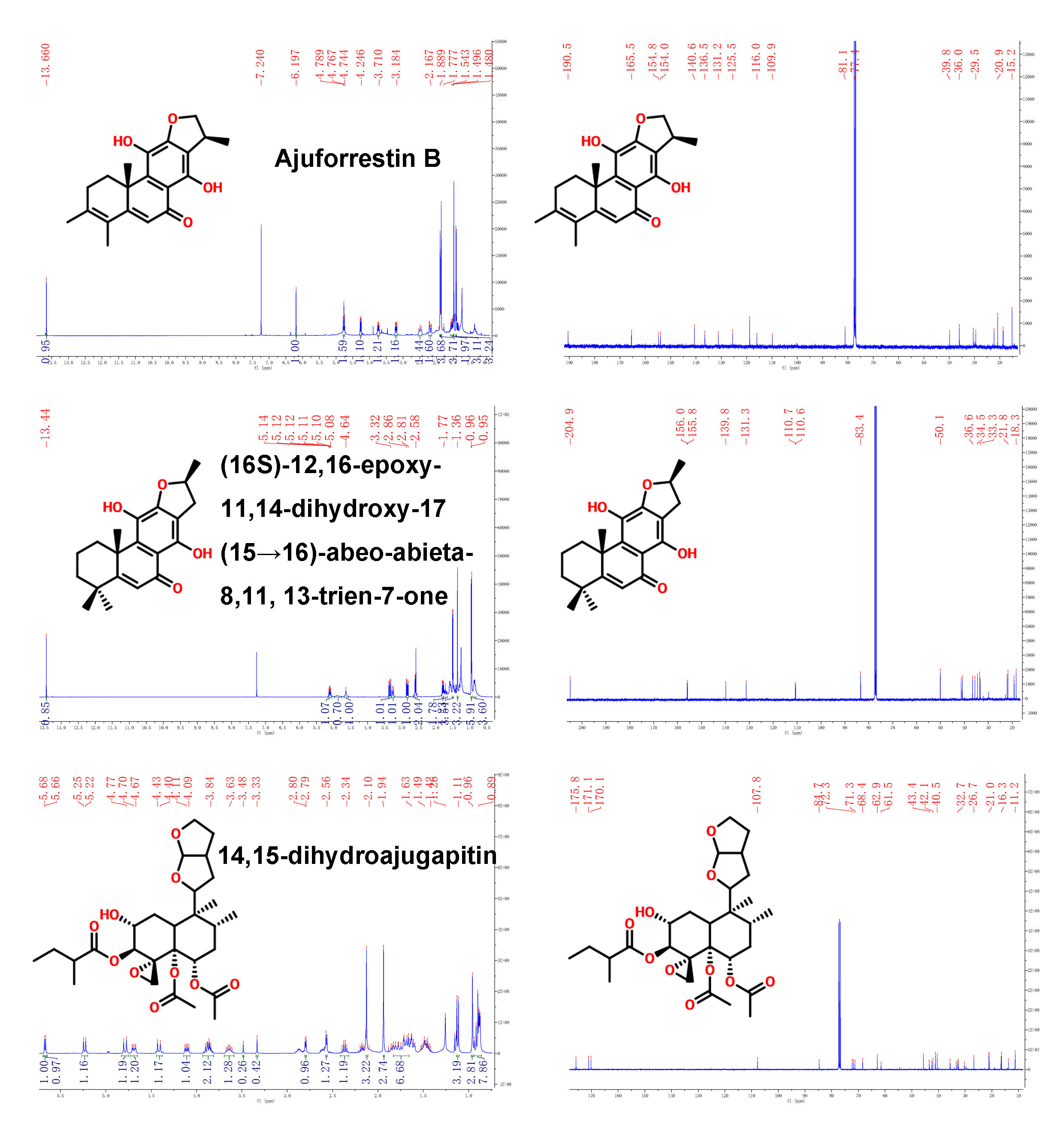


**Figure S2.** NMR identification of Ajuforrestin B, (16S)-12,16-epoxy-11,14- dihydroxy-17(15→16)-abeo-abieta-8,11, 13-trien-7-one, and 14,15-dihydroajugapitin.
